# GAIT ASSESSMENT IN ANKLE FRACTURES WITH SYNDESMOSIS LESIONS UNDERGOING SURGERY

**DOI:** 10.1590/1413-785220243206e281862

**Published:** 2025-01-10

**Authors:** Romero Montenegro Nery, Giovanni Dela Bianca de Ataide, Rafael Clark Gomes, Dallianny Gonçalves de Sousa Martins, Giovanni Ítalo Gomes de Almeida, Lucas Amaral Shizue Suassuna, Epitácio Leite Rolim-Filho

**Affiliations:** 1. Hospital Getulio Vargas, Departamento de Ortopedia e Traumatologia, do Hospital Getúlio Vargas, Recife, Pernambuco, PE, Brazil

**Keywords:** Ankle Fracture, Three-Dimensional Gait Assessment, Syndesmosis, Biomechanics, Fratura do Tornozelo, Avaliação Tridimensional da Marcha, Sindesmose, Biomecânica

## Abstract

Introduction: The three-dimensional evaluation of patients in the gait laboratory is a diagnostic method that is gaining ground in various orthopedic pathologies and, in the case of ankle fractures, can more accurately detail the degree of joint limitation. Objective: To present the importance of laboratory gait studies in the postoperative period of ankle fractures associated with syndesmosis ligament injuries, increasing the arsenal for assessing whether the surgical approach and outcome were satisfactory. Methods: Case series of 13 patients who underwent surgical treatment for ankle fractures associated with syndesmosis injuries, evaluated postoperatively in the gait clinic using the BTS GAITLAB hardware program. Kinetic and kinematic data using a three-dimensional movement system were collected and analyzed. Results: Alterations were found in the Temporal and Spatial Parameters and in the Statistical Angles of the lower limb joints, comparing the operated limb with the non-operated limb. Conclusion: The results of the study suggest that, despite subtle variations between the limbs assessed, the program was able to identify these differences in a significant way, demonstrating that gait assessments bring great benefits in understanding biomechanical limitations, and make more effective and individualized rehabilitation protocols possible. *Level of evidence IV, Case series.*

## RESUMO

Introdução: A avaliação tridimensional dos pacientes no laboratório de marcha é um método diagnóstico que vem ganhando espaço nas várias patologias ortopédicas e, no caso das fraturas do tornozelo, pode detalhar com mais precisão o grau de limitação articular. Objetivo: Apresentar a importância do estudo da marcha em laboratório no pós-operatório das fraturas do tornozelo assoc iadas a lesões dos ligamentos da sindesmose, aumentando o arsenal de avaliação se a conduta e o resultado cirúrgico foram satisfatórios. Métodos: Série de casos de 13 pacientes submetidos a tratamento cirúrgico das fraturas do tornozelo associada a lesão da sindesmose, avaliados no pós-operatório no ambulatório de marcha com uso do programa Hardware BTS GAITLAB. Dados cinéticos e cinemáticos através de sistema tridimensional de movimento foram coletados e analisados. Resultados: Alterações foram encontradas nos Parâmetros Temporais, Espaciais e nos Ângulos Estatísticos das articulações dos membros inferiores, comparando o membro operado com o não operado. Conclusão: Os resultados do estudo sugerem que, apesar de variações sutis entre os membros avaliados, o programa foi capaz de identificar essas diferen&c cedil;as de maneira significativa, demonstrando que avaliações de marcha trazem grandes benefícios no entendimento das limitações biomecânicas, e tornam possíveis protocolos de reabilitação mais efetivos e individualizados. *Nível de evidência IV, Série de casos.*

## INTRODUCTION

 Ankle fractures are a common type of fracture, accounting for approximately one in ten orthopedic fractures, with lateral malleolus injury accounting for 55% of cases. In most cases, it is mainly caused by torsional trauma, and when it is associated with injury to the distal tibiofibular joint (syndesmosis), the chances of some kind of sequel increase, even when treated correctly. In this sense, the better and more assertive the assessment in the pre-and postoperative period, the greater the chances of successful treatment and functional return of the patient. ^1^ ^-^ ^5^

 Initially, it is necessary to understand the complexity of this joint, which is composed of a synovial fitting involving the articular surface of the tibia (ankle and talus), working together with the subtalar joint, that is, it acts as an improved hinge that allows plantar flexion, and flexion, sliding and rolling on the dorsal side. In addition, the ankle is bounded by three lateral ligaments and a strong medial delta ligament. Therefore, this is a complex area with many potential sites of injury. ^3^ ^,^ ^4^

 In recent years, new diagnostic imaging methods have been proposed. These methods aid in both the pre- and postoperative periods of these lesions. The three-dimensional assessment of patients in the gait laboratory is a diagnostic method that has been increasingly used for various orthopedic conditions. In the case of ankle fractures, it can provide a more detailed evaluation of the extent of joint limitations through gait analysis. Evaluating the principles of gait analysis, with a particular focus on the foot and ankle, has revealed some previously misunderstood concepts. ^5^ ^,^ ^6^

 The study of gait has contributed positively in the various pathologies of the foot and ankle segment, with studies showing its postoperative relevance mainly in patients undergoing ankle arthrodesis and ankle arthroplasty surgeries, ^7^ ^-^ ^9^ providing accurate assessments to intervene more effectively, with evident improvement in gait quality. Such contributions are fundamental in the anatomical restoration and stabilization of tibiofibular syndesmosis, preventing a chronic pattern characterized by persistent ankle pain, functional disability, and early osteoarthritis. ^10^ ^-^ ^14^

Despite the importance of this type of assessment, studies that show the quality of gait after ankle fractures with ligament injury are still scarce in the literature. For this reason, the aim of this study was to evaluate the gait of patients with ankle fracture associated with syndesmosis ligament injury submitted to surgical treatment, through three-dimensional biomechanical analysis.

## MATERIALS AND METHODS

### Study Design

This is a prospective case series conducted on patients with ankle fractures associated with syndesmosis injury who underwent surgical treatment for both the fracture and ligament injury. Patients were recruited consecutively at the UNIMED, Português and Santa Joana hospitals. They were followed at the Movement Analysis Laboratory of the Instituto Rolim, in Pernambuco-Brazil, from June 2020 to August 2022. This study was approved by the Research Ethics Committee of the Universidade Federal de Pernambuco (no. 5,841,495) and followed the international declaration of intent to treat.

### Eligibility criteria

Patients, both male and female, aged 25 to 62, who had undergone appropriate postoperative physiotherapy within 6 to 24 months were included. Patients who did not undergo a proper gait examination and those with other orthopedic issues in the lower limbs, upper limbs, or spine were excluded.

### Surgical intervention

The recommended surgical technique for the study involved open reduction of the fracture using the OA technique, with osteosynthesis of the malleoli using plates and/or screws. To treat syndesmosis lesions, we performed fixation using either syndesmotic buttons or 3.5 mm screws.

### Variables

#### Clinical and radiological evaluation

Patients were assessed using a questionnaire containing clinical data and a validated functional assessment tool, AOFAS. In addition, we performed X-ray examination to assess post-surgical alignment.

#### Anthropometric assessment

Static anthropometric data include distance between the anterior superior iliac spines (ASIS), pelvic girdle depth, length of the lower limbs, diameter of the knees, and diameter of the ankles. After collecting the static data, we also measured the dynamic data, such as the range of motion of the hips, knees, ankles, and feet.

#### Gait movement: reflective markers

**Figure 1.**
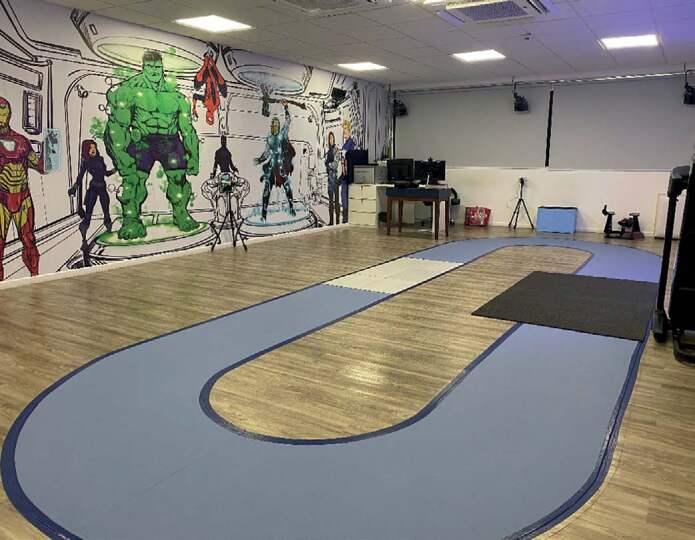
Center for Rare Diseases of Pernambuco (Rarus) and Movement Analysis Laboratory of Instituto Rolim.

**Figure 2.**
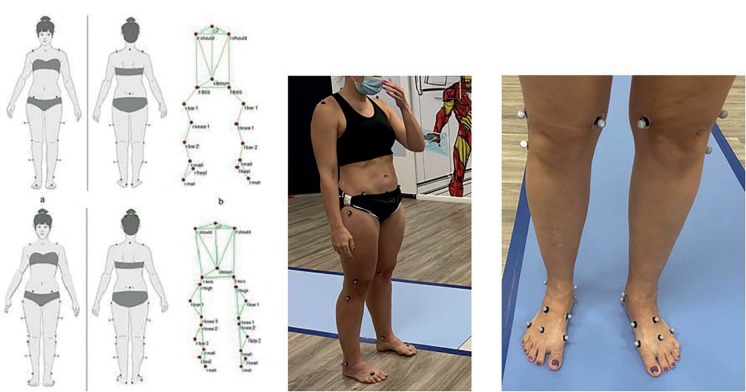
Reflective markers for motion capture: painless stickers on the patient’s skin.

 Kinetic and kinematic studies were conducted in the gait laboratory using BTS GAITLAB Hardware, which includes 10 cameras and 6 force platforms, along with markers (sensors) as shown in Figure 1 and 2 . 

 After gathering the anthropometric data, the BTS-Gaitlab program was established, as previously described. The modified Helen Hayes protocol has been implemented. This protocol utilizes a specific set of markers (sensors), as described by Kadaba et al. ^15^ (15). Reflective markers for motion capture were placed on anatomical sites defined by the chosen protocol and attached to the patient’s skin using painless adhesives. 

After placing the markers, the patients were asked to perform two different tasks.

**Static socket** (Standing) - patients were instructed to maintain an orthostatic position for about 5 seconds, with the feet aligned at the top of the power platform as shown. This protocol calculates the patient’s static pose joint angles and creates a three-dimensional reconstruction. During data processing, a report is generated that displays a table containing the angular values alongside the normative data. 

**Dynamic take** - The patients were instructed to walk naturally on the lab’s track, equipped with six digital 3D force platforms that capture their reaction forces to the ground. Each patient performed approximately 18 repetitions. Reflective markers are tracked by 10 high-resolution, high-frequency infrared cameras, providing data on joint position and movement during walking. The data is recorded and transmitted to a computer using Bluetooth technology. 

Each complete examination lasted on average one to two hours. It was necessary to use bathing suits, however, if patients chose, they could use other garments, such as shorts and T-shirts. Before the sessions began, the system was properly calibrated.

**Figure 3.**
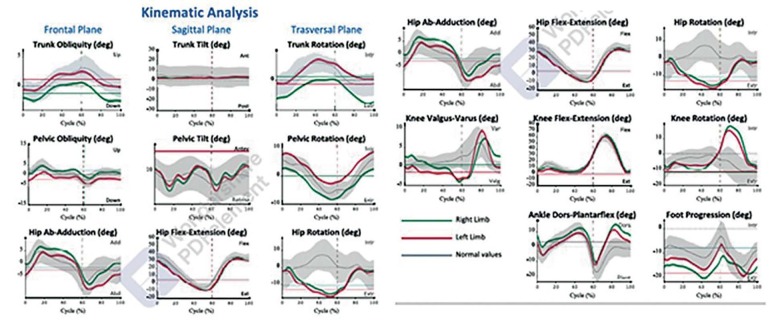
BTS-GAITLAB software has several functions that allow you to view in detail the data obtained in multimedia: spatio-temporal parameters, kinematics and kinetics of joints in different phases of the gait cycle.

 After collecting these kinetic and kinematic data, the Helen Hayes protocol was used automatically to process the examinations, which allows evaluating the joint movements of the lower limbs. Sessions were automatically filtered by protocol *Rep_Gait_Consistency* , obtaining an average gait parameter. The BTS-GAITLAB software offers various functions that allow detailed visualization of the collected data and the application of filters to ensure analysis accuracy. A multimedia report includes spatio-temporal parameters, kinematics and kinetics of the joints evaluated in the different phases of the gait cycle ( Figure 3 ). 

**Figure 4.**
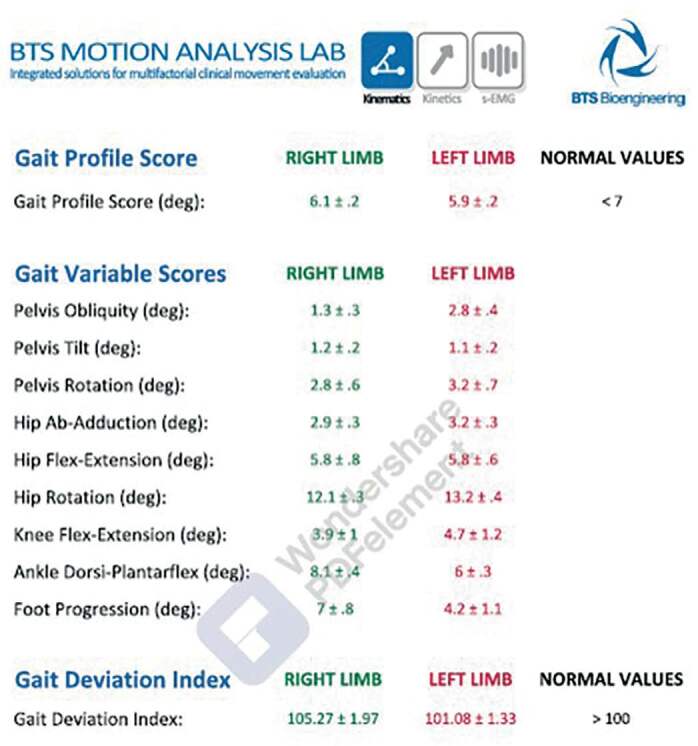
Gait quality analysis indices expressed as a percentage of the gait cycle: temporal, special and static.

 Some variables were collected and considered important: cadence (number of steps / min), speed (m / s), average speed (percentage of Height/s), step length (expressed as a percentage of the gait cycle), stride length (m), STEP width (m), support phase (expressed as a percentage of the cycle) swing phase (expressed as a percentage of the gait cycle), double Support (time when both feet were in contact with the ground, expressed as a percentage of the gait cycle), single support (expressed as a percentage of gait cycle), stride Time (s), support Time (s) and Swing Time(s) ( Figure 4 ). 

For the assessment of ankle and foot deviations, the dorsiflexion and plantarflexion angle of the ankle, as well as the foot progression angle, were primarily evaluated. Gait deviation index (GDI) and gait profile score (GPS) were used as gait quality analysis indices. These analyzed variables were compared with normal data in order to obtain a complete understanding of the possible movement compensation activities in the different anatomical planes and identify possible treatments for dysfunctions presented by patients.

 The Gait Deviation Index or GDI is a measure of general gait pathology. ^16^ It was developed from the kinematic data of a large number of walking strides to derive a set of mutually independent joint rotation patterns that efficiently describe gait. These patterns are called gait characteristics. A GDI value ≥ 100 indicates a subject whose gait characteristics are statistically indistinguishable from the gait characteristics of a given control group. In other words, a GDI value ≥ 100 indicates a normal gait. 

 The Gait Profile Score (GPS) and the Gait Variable Score (GVS) are two indices that summarize the overall quality of the patient’s gait kinematics. These indices facilitate the comparison of pathological and normal gait. The Gait Profile Score (GPS) is calculated as the Euclidean distance between the kinematic characteristics of the patient and the corresponding normative characteristics, for the entire gait cycle. GPS values greater than 7 degrees indicate a compromised gait pattern. ^17^

The Gait Variable Score (GVS) is defined as the mean square root of the difference between a single gait characteristic and the corresponding mean gait characteristic for people without gait pathology. GVS is calculated for each gait characteristic, and the results are presented in a specific table. This table provides useful information to understand which variables are contributing to a high Gait Profile Score (GPS).

After all this process, the Final report is created using the specific protocol. This report contains the average spatio-temporal parameters of all selected trials. Synthetic indices that summarize the overall quality of the patient’s gait make it easy to compare the pathological gait with the normal gait, which is well covered in the examination result. The graphs of kinematic and kinetic analysis are also presented. The unit of measurement used in the graphs is the degree (Y-axis) and the percentage of the March cycle (x-axis). The mean curves for each limb (green for the right lower limb and red for the left) are plotted against the normative data (in gray).

## RESULTS

### Sample data

 The data describing the demographic characteristics of the 13 eligible patients showed that most were female 9 (69.2%), with a mean weight of 79.39 kg and a mean height of 1.68 m ( Table 1 ). 

 The data from the examinations are presented in Table 2 . It is evident that the left side was the most frequently operated side, accounting for 61.5% of the total. According to the AO classification, Type B was the most frequent (69%), followed by Type C (31%), with no cases of Type A. In patients with Type B lesion, most were in Stage 2 and 3 (44% in each) and in Type C, 50% were in Stage 1 and 50% in Stage 2. In the LH classification, most patients were diagnosed with external supination-rotation (53.8%), 23.1% with external pronation-rotation and 23.1% with pronation-abduction. The mean follow-up time of patients after surgery and until examination was 12 months (minimum = 7 months; maximum = 19 months). 

**Table 1.** Demographic characteristics of the sample.

Variable	N (%)
Gender	
Female	9 (69.2)
Male	4 (30.8)
Age (years) mean standard deviation) (±SD)	40.54 (12.18)
Weight (kg) (mean ± SD)	79.39 (13.98)
Height (cm) (mean ± SD)	168.92 (9.98)
Postoperative time (months) (mean ± SD)	12.78 (4.36)

**Table 2.** Variables observed in the examination of patients.

Variable	n (%)
Side operated	
Right	5 (38.5)
Left	8 (61.5)
Classification AO	
44-A1	0 (0.0)
44-A2	0 (0.0)
44-A3	0 (0.0)
44-B1	1 (7.7)
44-B2	4 (30.8)
44-B3	4 (30.8)
44-C1	2 (15.4)
44-C2	2 (15.4)
44-C3	0 (0.0)
Classification Lauge Hansen	
external supination-rotation	7 (53.8)
Supination-Adduction	0 (0.0)
external pronation-rotation	3 (23.1)
Pronation-Abduction	3 (23.1)
AOFAS score Average	89.92 (4.63)

### AOFAS

**Figure 5.**
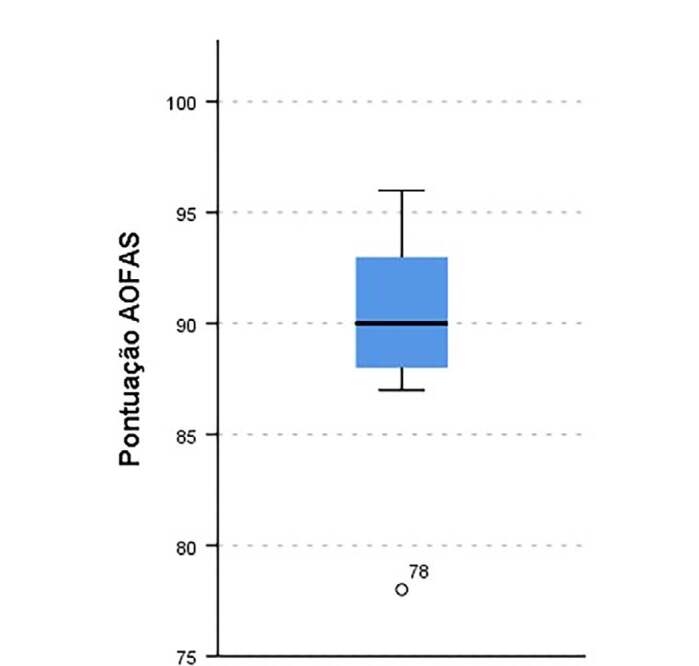
AOFAS chart.

 Through the AOFAS score it was possible to observe that most patients (84.6%) had AOFAS score classified as good, followed by excellent with 15.4%. No patient studied had an AOFAS score classified as reasonable or poor. In Figure 5 , it can be seen that the median score AOFAS was 90, the maximum value was 96, and the minimum value was 87 (disregarding the outlier value of 78). 

### Temporal parameters

Analyzing the temporal parameters, after the statistical analysis of the study patients, there was a difference between the operated limb and the contralateral side in the parameters support Time (p < 0.0001), Swing Time (p < 0.0001), Support Phase (p < < 0.0001), Swing Phase (p< 0.0001), Single Support Phase (p < 0.0001), and Double Support Phase (p = 0.0062).

The Swing Time and swing phase showed a significant variation on the operated side, with the normal parameter of 0.39 s (0.03). The Stride time and stride phase presented a reduction in time, both in relation to the contralateral limb and as a function of the parameters adopted as normal 0.93 s (0.04). The support Phase expressed in % also had a small increase with the right side 61.11% (1.75) and the left side 60.61% (1.27), with the normality value 57.97% (1.93). The double support phase and the Simple support Phase also showed significant differences, with a decrease in time in the left limb in the Simple Support Phase (34%) and an increase in both limbs in the Double Support Phase (D = 12%; E = 14%), compared to the standard value of 10%.

 Other temporal parameters also showed changes, such as average speed in meters per second, average speed in % of height per second and cadence, which is expressed in steps per minute. The average normal speed is 1.2 m/s, and in patients it was 1.08 m/s. The Average Speed (% Height/s) was 63.97% Height/s, the normal value being 80% Height/s. On the other hand, the cadence had an average value reduced from a normal value 1.2 m/s to 1.08 m/s. The data are shown in Table 3 . 

**Table 3.** Mean ± SD of the values observed in the variables of the physical examinations of the patients.

	Members		
	Right	Left	Default value (p vlue (v
Temporal Parameters			
Passing Time (s):	1.11 (0.09)	1.10 (0.09)	1.10 (0.899)
Support Time (s):	0.73 (0.01)	0.68 (0.02)	0.65 (<0.0001)
Balance Time (s):	0.34 (0.003)	0.43 (0.004)	0.44 (<0.0001)
Support phase (%):	65.38 (0.44)	60.89 (0.5)	58.98(<0.0001)
Swing phase (%):	*34.61 (1.75)	38.14 (1.27)	40.03(<0.0001)
Single support phase (%)	38.73 (0.40)	34.86 (0.44)	38.87(<0.0001)
Double support phase (%):	12.07 (0.17)	14.23 (0.73)	10.27 (0.0062)
Average speed (m/s):	1*.08 (0.13)		
Average speed (%height/s):	*63.97 (6.20)		
Cadence (steps/min):	*106.99 (8.17)		

### Spatial parameters

The spatial parameters showed few changes compared to the normal reference values, always within the limits considered normal. However, when comparing the operated limb with the contralateral one, a statistically significant difference was observed. Stride length, which is expressed in meters, had a slight increase overall, with an average of both the right (1.58 m) and left (1.48 m), with the average reference value being 1.13 m, with p < 0.0001. Stride length in % per height had a decrease in the overall value of the patients, with the normal value being 80 (10), in the right limb 65.29 (0.83) and left limb 63.58 (0.86) with p < 0.0001.

 The step length, expressed in meters, was the only parameter without significant difference between the limbs, with little variation in relation to the reference value, p = n. s. These values are expressed in Table 4 . 

**Table 4.** Mean ± SD of the values observed in the variables of the physical examinations of the patients.

	Members		
	Right	Left	P-Value
Spatial Parameters			
Stride length (m):	1.58 (0.27)	1.48 (0.28)	<0.0001
Stride length (%alt.):	64.29 (0.83)	63.58 (0.86)	<0.0001
Step length (m):	0.51 (0.16)	0.52 (0.15)	0.61
Step width (m):	0.09 (0.02)		

### Statistical angles

 Regarding the statistical angles (expressed in degrees), some small changes were observed in the pelvis, hip, knee, ankle and foot. Hip Ab-adduction (°) was the parameter that presented a significant difference between the operated and contralateral limbs (p = 0.017) the other data were not statistically different, as shown in Table 5 . 

Table 6 , which details the descriptive measures of the parameters of Gait Profile Score, Gait Variable Scores and Gait Deviation Index, it is possible to observe that neither the mean observed value of GPS nor the GDI were statistically different from the standard value. 

**Table 5.** Mean ± SD of the values observed in the variables of the physical examinations of the patients.

	Members (average and SD)		
	Right	Left	P-value
Static Angles			
			
Pelvic obliquity (degrees):	1.09(0.20)	1.09 (0.20)	> 0.99
Pelvic Tilt (degrees):	10.28 (1.78)	10.28 (1.78)	> 0.99
Pelvic rotation (degrees):	3.2 (2.98)	3.2 (2.96)	> 0.99
Hip Ab-adduction (°):	1.29 (0.50)	3.22 (0.15)	0.017*
Flexion-extension of the hip (°):	7.39 (5.00)	6.71 (5.54)	0.239
Hip rotation (°):	9.06 (3.8)	7.73 (2.73)	0.77
Flexion-extension of the knee (°):	0.90 (3.31)	0.77 (3.95)	0.82
Plantar dorsiflexion of the ankle.(°):	4.01 (0.12)	4.89 (0.33)	0.063 (or 0.22)
Foot progression (°):	9.18 (0.7)	9.30 (0.8)	0.89

**Table 6.** Mean (standard deviation) of the values observed in the variables of the physical examinations of the patients.

	Member		
Variables	Right	Left	Default value (p value)
Gait Profile Score			
Gait Profile Score (deg):	7.92 (0.8)	7.69 (1.2)	< 7 (p = 0.643)
Gait Variable Scores			
Pelvic obliquity (degrees):	1.74 (0.64)	2.22 (0.80)	
Pelvic Tilt (degrees):	4.08 (3.01)	4.06 (3.03)	
Pelvic rotation (degrees):	2.99 (0.68)	2.95 (0.42)	
Hip Ab-adduction (°):	3.23 (1.41)	2.91 (1.29)	
Flexion-extension of the hip (°):	5.18 (2.37)	4.67 (2.29)	
Hip rotation (°):	9.84 (3.84)	10.07 (3.94)	
Flexion-extension of the knee (°):	5.22 (1.51)	5.56 (2.59)	
Plantar dorsiflexion of the ankle.(°):	5.26 (1.19)	6.00 (1.30)	
Foot progression (°):	7.16 (4.88)	6.48 (4.77)	
Gait Deviation Index	90.92 (2.6)	89.50 (2.8)	> 100 (p = 0.034)

## DISCUSSION

Adequate treatment of syndesmosis complex lesions is challenging but necessary to avoid malreduction, which can alter the kinematics of the Tibio-fibular joint and lead to chronic instability, cartilage damage, and early osteo-arthritic changes of the ankle joint. Therefore, the accuracy and maintenance of syndesmosis reduction are considered essential in the treatment of ankle fractures with concomitant syndesmosis injury. The postoperative parameters of physical examination and imaging tests give us an insight into what degree of limitation the operated patient may have, but not in a dynamic way.

The study of gait in fractures of the lower limbs has been gaining ground, evaluating the kinetics and kinematics of patients. Observing some specific works of patients with ankle fracture associated or not with syndesmosis injury, several small changes in gait pattern were observed.

 Researchers compared gait patterns among patients treated for ankle fractures with those of healthy individuals. They analyzed 18 patients with ankle fracture using PROM and gait, with a multisegmental foot model (modified Oxford foot model) with at least one year postoperatively. Twelve patients had lateral uni-maleolar fracture and six had tri-maleolar fracture, and all were treated with open reduction and internal fixation. The results were compared with those of healthy subjects and the contralateral leg. The study found lower flexion / extension between the hindfoot and tibia in the fracture group compared to healthy subjects during support, and lower ROM (flexion/extension) in the swing phase compared to the uninjured side. They found that the Olerud and Molander ankle score (OMAS) questionnaire correlated moderately to moderately with kinematic parameters in the sagittal plane during the swing (flexion/extension) phase. ^19^ These changes were also observed in the present study. 

 During early rehabilitation, ankle fracture patients may develop asymmetry of trunk movement in the vertical direction accompanied by slower gait speed and cadence and shorter step lengths, which may contribute to muscle imbalances and potential injury. Thus, appropriate rehabilitation strategies should be employed for these patients. ^18^ ^-^ ^20^

 Losch et al. ^21^ analyzed gait in 20 patients with surgically treated ankle fracture one year after the operation and compared the results with those of 20 healthy adults. They found lower flexion / extension in the ankle joint, lower speed, and shorter stride length in the injured group compared to healthy individuals. However, they found no significant correlation between kinematic parameters and Patient Reported Outcome Measures (PROM). 

 The work of Segal et al., ^22^ evaluated some parameters such as step length, walking speed and plantar pressure in patients operated on ankle fractures. The 41 patients with ankle fracture were divided into uni-maleolar fracture (n = 12), bi-maleolar fracture (n = 15) and tri-maleolar fracture (n = 15). The results were compared with the gait of 72 healthy subjects. There were significant differences in all parameters, primarily walking speed and step length. Patients with uni-maleolar fracture performed better than others with bi-maleolar or tri-maleolar fractures. 

 Hancock et al. ^23^ , and that of Egol et al. ^12^ , also evaluated the functional outcome after ankle fractures, but reported different results regarding the severity of the fracture and the functional outcome. Researchers saw that individuals with uni-or bi-malleolar ankle fractures had better functional outcome than patients with tri-malleolar fractures, based on the OMAS and Lower Extremity Functional Scale (LEFS). In contrast, the work of Egol et al. They concluded that the type of fracture had no influence on the functional outcome after ankle fracture surgery, according to the Orthopedic Trauma Association (OTA) system and the Lauge-Hansen system. 

Various gait changes occur after ankle fracture, including reduction in stride length, Swing Time, single support time, stride length, cadence, speed, and a fore-foot exit time on the affected side. In addition, the symmetry of trunk movement (especially vertical) is significantly reduced after ankle fracture.

 The differences in the kinematic profile of the gait of the lower limbs of patients recovering from ankle fracture compared to healthy controls, were evaluated in a study. In addition, we asked whether the profile would be different between the groups of fracture severity. A total of 48 patients participated in the prospective case-control study. The gait of 24 patients recovering from an ankle fracture injury and 24 healthy paired controls was examined using an inertial measurement unit sensor system. The following gait parameters were evaluated: knee range of motion (ROM) during the swing phase, maximum knee flexion angle during support, thigh and calf ROM, and stride duration. Statistically significant differences were found between the ankle fracture group and the control group for all parameters. Patients with ankle fracture had lower ROM of the knee during the rocking phase compared to the control group (mean ± standard deviation 43.0° ± 15.5° compared to 66.7° ± 5.1°, respectively (p < 0.001). The maximum knee flexion angle during support was lower in patients with ankle fracture than in the control group (mean ± standard deviation 10.5° ± 6.1° compared to 21.2° ± 4.5°, respectively; p < 0.001). Ankle fracture patients also had lower thigh and calf ROM angles (p < 0.001) and longer stride duration (p < 0.001) compared to the control group. No statistically significant differences were found between the severity groups. These results suggest that gait kinematic characteristics vary between healthy people and patients recovering from an ankle fracture during the short period after the injury ^24^ . 

The hypothesis was that patients after ankle fracture surgery had less ankle flexion/extension compared to healthy subjects and that fracture severity had a significant influence on kinematics and patient satisfaction. Thirty-three patients (n = 33 feet) operated for ankle fractures were recruited. Ankle kinematics were analyzed using the Oxford Foot, and the results were compared with a healthy control group of the same age (n = 11 patients, 20 feet). In addition, patients were divided by fracture classification (severity) and kinematic results were correlated with PROM and radiographic findings. Patients treated for ankle fracture showed lower walking speed (p < 0.001) when asked to walk preferably at normal speed. When compared at equal speed, significantly lower range of motion (ROM) between the hindfoot and tibia in the sagittal plane (flexion/extension) during loading and push-off (p = 0.003 and p < 0.001) was found in patients after ankle fractures compared to healthy subjects. Lower ROM and worse PROM outcomes were found for patients with tri-malleolar fractures of the ankle. There was a significant correlation between ROM (flexion/extension) during the push-off phase and physical functioning SF-36 (r2 = 0.403, p = 0.027) and SF-36 general health (r2 = 0.473, p = 0.008). Fracture severity was significantly correlated with ankle flexion/extension ROM during the load and thrust phases (r2 = -0.382, p = 0.005 and r2 = -0.568, p < 0.001) and was also significantly correlated with PROM. This study found that patients with ankle fractures had significantly altered ankles in kinematics compared to healthy subjects.

Several parameters are evaluated in the gait study, with subtle or slightly more exacerbated changes, which was also evaluated in the present study. In the Temporal Parameters it is noted that the greatest changes were in the Average Speeds and Cadence. The spatial parameters showed few changes compared to the normal reference values, always within the limits of normality. Regarding the statistical angles, some small changes were observed in the pelvis, hip, knee, ankle and foot, without much relevance.

The descriptive measurements of the gait Profile Score, Gait Variable Scores and Gait Deviation Index parameters allow to observe that neither the mean observed value of GPS nor the GDI were statistically different from the standard value.

Evaluation of markers with the use of gait movements can provide an objective characterization of gait changes after ankle fracture. This assessment is important not only in clinical practice to assess patient performance, but also in clinical research as a reference point for evaluating existing or new rehabilitative interventions, and can provide an objective characterization of gait changes after ankle fracture.

The kinetics and kinematics of the ankle used in this research showed to be efficient in verifying the behavior of the individual during the main phases of gait. The data collected showed consistency with the expected pattern of normal gait for all volunteers, in an absolute analysis. Further research should be carried out with a larger number of samples, comparing specific groups, and investigating intervening variables of the gait cycle.

The study was greatly limited by the small number of patients evaluated. Despite the fact that it is a relatively common fracture treated in emergencies, the analysis in the Gait Laboratory requires a relatively long time to perform the examination, and many patients refuse to perform it for this reason. In addition, the collection of the examination requires a health professional who knows how to handle the sensors and use the specific program. It should also be noted that Gait Laboratories are scarce in the country because they are very expensive, in addition to interpreting the three-dimensional data is quite difficult and requires trained professionals.

The results presented in this study justify the use of gait in clinical practice and encourage the development of intervention methods that emphasize function. Gait markers positively interfere with gait locomotor function in patients with ankle fracture associated with syndesmosis and enhance the retention of skills developed in training in the medium term.

## CONCLUSION

The results of the study suggest that, despite subtle variations between the evaluated limbs, the program was able to identify these differences significantly, demonstrating that gait evaluations after ankle fracture surgeries with syndesmosis fixation, with a biomechanical program, will bring great benefits both to understand the possible limitations that the patient may present, and to form earlier rehabilitation protocols and consequently improve short-and long-term results.
